# The Magistral Phage

**DOI:** 10.3390/v10020064

**Published:** 2018-02-06

**Authors:** Jean-Paul Pirnay, Gilbert Verbeken, Pieter-Jan Ceyssens, Isabelle Huys, Daniel De Vos, Charlotte Ameloot, Alan Fauconnier

**Affiliations:** 1 Laboratory for Molecular and Cellular Technology, Queen Astrid Military Hospital, Bruynstraat 1, 1120 Brussel, Belgium; gilbert.verbeken@mil.be (G.V.); danielmarie.devos@mil.be (D.D.V.); 2 Bacterial Diseases, Unit Antibiotic Resistance, Scientific Institute of Public Health, Rue Engelandstraat 642, 1180 Brussel, Belgium; pieterjan.ceyssens@wiv-isp.be; 3 Department of Pharmaceutical and Pharmacological Sciences, KU Leuven, O&N2, Herestraat 49, Box 521, 3000 Leuven, Belgium; isabelle.huys@pharm.kuleuven.be; 4 Federal Agency for Medicines and Health Products, Place Victor Horta 40/40, 1060 Brussels, Belgium; charlotte.ameloot@fagg-afmps.be (C.A.); alan.fauconnier@invivo.be (A.F.); 5 Culture In Vivo ASBL, rue du Progrès, 4, boîte 7, 1400 Nivelles, Belgium

**Keywords:** antibiotic, antimicrobial resistance, magistral preparation, compounding pharmacy, phage therapy, regulatory framework, personalized medicine

## Abstract

Since time immemorial, phages—the viral parasites of bacteria—have been protecting Earth’s biosphere against bacterial overgrowth. Today, phages could help address the antibiotic resistance crisis that affects all of society. The greatest hurdle to the introduction of phage therapy in Western medicine is the lack of an appropriate legal and regulatory framework. Belgium is now implementing a pragmatic phage therapy framework that centers on the magistral preparation (compounding pharmacy in the US) of tailor-made phage medicines.

## 1. The Age of the Superbug

On 21 September 2016, the UN General Assembly convened a meeting on antimicrobial resistance (AMR) at the UN headquarters in New York. It was only the fourth time the General Assembly addressed a health emergency. This high-level meeting resulted in a UN resolution focused on combatting the AMR health threat. World leaders acknowledged that global AMR poses a fundamental long-term threat to human health, the production of food, and sustainable development. Based on scenarios of rising drug resistance for six pathogens, experts estimated that by 2050 the burden of AMR could rise to 10 million people dying every year and an economic cost of $100 trillion [1].

Commercial antibiotics that are currently used in public health, animal, food, agriculture and aquaculture sectors, are immutable chemicals that are based on natural antibiotics produced by soil bacteria or fungi to—depending on their concentration—either combat competitors, communicate with other organisms, or act as pleiotropic effectors of metabolic pathways. It was, therefore, to be expected that bacteria would be extremely proficient at evolving resistance to such antibiotics, especially when these are excessively and often unnecessarily used. Selective pressures imposed by humans have resulted in the emergence of “superbugs”, or bacteria that are resistant to virtually all commercial antibiotics. Experts fear that society could return to a pre-antibiotic era, when simple infections could wipe out entire populations and surgical interventions were life threatening. Today, it seems that all “easy” antibiotics have been exploited and industry has been reluctant to put new efforts into the discovery and development of new classes of antibiotics. These are expensive to develop and are bound to offer a poor return on investment as they are only taken for a short period and their use is likely to be restricted in the future.

Therefore, the UN committed to work at national, regional and global levels to support the development of new antimicrobial agents and therapies [2].

## 2. Phage Therapy

One of the promising “new” treatments that is increasingly highlighted—*inter alia* during the recent UN General Assembly—is phage therapy, the therapeutic use of bacteriophages (phages in short)—the viruses of bacteria—to treat bacterial infections [3]. Since time immemorial, phages control their hosts, the bacteria, on our planet. When discovered in the early twentieth century, they were immediately applied in medicine. It soon appeared that phages are exquisitely host-specific. Most phages can only lyse a subset of a bacterial species. Physicians must thus first know which bacteria cause the infections before they can treat the patients.

As could be expected, it was shown that bacteria could also evolve to evade phage infection, even when potent phages are applied simultaneously [4]. However, the main advantage of phages over antibiotics is their ability to mutate at least as fast as their hosts, enabling them to evolve new infectivity and thus regain the “upper hand” over bacteria. Bacteria and phage are thus involved in a continuous arms race of co-evolving infectivity and defense mechanisms.

The advent of broad-spectrum antibiotics, which target a wide range of bacterial infections and could thus be used empirically, heralded the decline of phage therapy in the Western world. The success stories of the many phage applications in the past, mainly on the east side of the Iron Curtain, where phage therapy remained an established treatment, together with the increasing number of virtually untreatable bacterial infections, has created a growing demand for phage therapy. Some successful intravenous applications of phages to treat terminally ill patients in the Western world have recently been published in the scientific literature [5,6].

### The Promise of the Phage Therapy Medicinal Product

At their reintroduction in the Western world, phage preparations were classified as medicinal products (European Union) or drugs (US), based on the literal implementation of definitions. Namely, any substance presented as having properties for treating or preventing disease in human beings is considered to be a medicinal product or a drug. As a result, a large body of costly and time-consuming requirements and procedures for manufacturing and for obtaining marketing authorization for medicinal products (drugs in the US) for human use were imposed on phage therapy medicinal products (PTMPs).

On the one hand, it turns out that the established pharmaceutical industry is not interested in PTMPs, mainly because of limitations in intellectual property protection of natural entities such as genes or phages and because of phage specificity and bacterial resistance issues, which compromise widespread and long-term use of immutable pre-defined PTMPs. On the other hand, it is becoming clear that medicinal product provisions, which were originally developed to cater for widely used and mass-produced chemical molecules such as aspirin and antibiotics, are not compatible with sustainable (non-empirical) or customized phage therapy approaches in which phages need to be selected and produced ad hoc [7]. Pre-defined PTMPs, could make it through the medicinal product funnel, but such preparations are less flexible to deal with changes in the incidences of infecting bacterial species in certain settings or geographical areas, or with the emergence of mutated bacterial strains. The long-term use of immutable PTMPs is also bound to elicit considerable bacterial phage resistance, although not much is known about the rate at which this would occur in clinical settings. Overall, the efficacy of PTMPs is likely to decrease over time and they would need to be regularly adapted and re-approved for use.

Some of these issues crystallized during PhagoBurn (www.phagoburn.eu), the first major trial under modern medicinal product regulatory standards in the European Union [8]. Cocktails of 12 and 13 phages were needed to ensure a certain activity against a collection of *Pseudomonas aeruginosa* and *E. coli* isolates, respectively. Manufacturing of one batch of the investigational products ended up taking 20 months and the largest part of the study budget. In addition, phage specificity issues hampered the recruitment of patients. Because each of the two study products, which couldn’t be applied simultaneously, targeted only one of the multiple bacterial species that are known to (simultaneously) infect or colonize burn wounds, physicians were reluctant to include patients [8]. Regardless of the final clinical outcome of PhagoBurn, the preliminary phase of the study showed at least that dedicated and realistic production and documentation requirements are urgently needed to enable the timely supply of secure phage preparations. This would enable clinicians to conduct the desperately needed safety and efficacy studies and to deal with urgent individual or local infection issues or public health threats (e.g., the 2011 *E. coli* O104:H4 outbreak in Germany).

Meanwhile, sporadic phage applications are carried out in the West, often under the umbrella of Article 37 (Unproven Interventions in Clinical Practice) of the Declaration of Helsinki (www.wma.net). In addition, several European and US patients suffering from chronic, extremely resistant or difficult to treat bacterial infections are known to have travelled to a phage therapy center in Tbilisi, Georgia (www.eliavaphagetherapy.com, www.phagetherapycenter.com), for treatment.

## 3. Enter the Magistral Phage

On 5 July 2016, during a meeting of the Belgian Chamber of Representatives and in response to two parliamentary questions related to the implementation of phage therapy [9], the Belgian Minister of Social Affairs and Public Health acknowledged that phage therapy has no specific regulation in Europe and that there is a consensus that phage preparations are medicinal products. However, according to the Minister it is difficult to determine whether we should deal with industrially-prepared medicinal products or rather with magistral preparations, the former being subject to constraints related to their production and marketing authorization, unlike the latter.

### 3.1. Magistral Preparations

In European and Belgian law, the notion of a magistral preparation (compounded prescription drug product in the US) is defined as “any medicinal product prepared in a pharmacy in accordance with a medical prescription for an individual patient” (Article 3 of Directive 2001/83 and Article 6 quater, § 3 of the Law of 25 March 1964). Magistral preparations are mixed from their constituent ingredients by a pharmacist (or at least under his/her supervision), for a given patient according to a prescription by a physician and following the technical and scientific standards of the pharmaceutical art. The magistral formula is a practical way for a medical doctor to personalize patient treatments to specific needs and to make medications available that do not exist commercially. Some medicines, such as natural hormone combination products and allergens, are not produced by commercial manufacturers because they lack patent protection and hence return on investment for pharmaceutical companies, but are actually delivered as magistral preparations. Owing to the emergence of innovative medicines for rare diseases or for personalized therapies, magistral preparations are increasingly in demand.

### 3.2. The Belgian Magistral Phage Medicine Strategy

The Community code leaves the door open for some flexibility to implement certain national solutions relating to medicines for human use [10]. As such, the Belgian Minister of Public Health asked the Federal Agency for Medicines and Health Products (FAMHP, the Belgian competent authority for medicines) to help set up a national strategy for magistral phage medicines. In general, active ingredients of magistral preparations must meet the requirements of the European Pharmacopoeia, of the Belgian Pharmacopoeia or of an official pharmacopoeia [10]. If no such document exists, then the active ingredients must be authorized by the Minister of Public Health, following a favorable opinion of the national Pharmacopoeia Commission [10]. In addition, non-authorized ingredients may also be used in magistral preparations, providing that they are accompanied by a certificate of analysis issued by a Belgian Approved Laboratory [10]. The so-called “Belgian Approved Laboratories” are quality control laboratories which are granted an accreditation by the Belgian regulatory authorities. This status allows them to perform the batch release testing of medicinal products. This national accreditation is equivalent to—and gradually replaced by—the GMP certification for the batch release testing of medicinal products. Belgian Approved Laboratories can be either private (e.g., subcontractor of the pharmaceutical industry) or partially or entirely public (e.g., academic laboratories and scientific institutes). Some of them belong to the European Official Medicines Control Laboratories (OMCL) network, which is made up of independent public laboratories that have been appointed by their respective national authority.

The option of the “non-authorized ingredient” was chosen in this case because of the enormous variety of phages that could qualify as active ingredients and should then, each individually, obtain an authorization issued by the Minister of Public Health [9]. The Scientific Institute of Public Health was identified as a suitable Belgian Approved Laboratory for issuing valid certificates of analysis for batches of phage active ingredients. Although the standard procedure for unauthorized active ingredients only involves the medical doctor, his patient, the manufacturer of the active substances, the approved laboratory and the pharmacist, it was decided—in joint consultation and because of the innovative and very specific character of phage therapy—to involve the FAMHP in the elaboration of a Belgian magistral phage medicine procedure.

In practice, and to consolidate the opening left by the Minister of Public Health, a formal question and answer session was initiated between the military hospital and the FAMHP within the context of the existing national Scientific-Technical Advice (STA) procedure. On 26 October 2016, it was formally agreed that natural phages whose derivative finished products are not fully compliant with the requirements relating to medicinal products for human use (Directive 2001/83), and for which there is no monograph in an official pharmacopoeia, can be processed by a pharmacist as active pharmaceutical ingredients (APIs) in magistral preparations, providing compliance to a number of logical provisions:

- Phages should be delivered in the form of a magistral preparation to a specific (nominal) patient.
- Magistral preparations should always be delivered under the direct responsibility of a medical doctor and a pharmacist.
- The relevant characteristics and qualities of the phage APIs should be defined in an internal monograph (prepared by the supplier).
- Before the pharmacist can use the unlicensed material, he/she must ascertain—based on certificates of analysis issued by a Belgian Approved Laboratory—that the raw materials conform to the provisions of the internal monograph.
- Even if not legally required, it is recommended that the supplier submits the monograph for assessment by the FAMHP.

**Figure 1:**
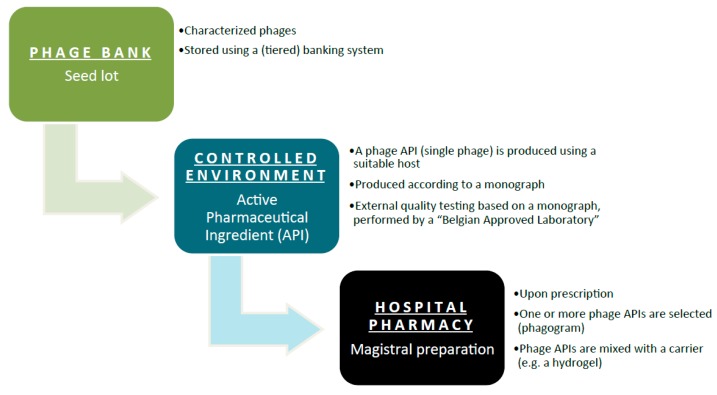
General flowchart of the magistral phage medicine process.

The general concept of the Belgian magistral phage medicine strategy is depicted in Figure 1. A single characterized phage seed lot is selected from a phage bank. To prevent the unwanted drift of properties resulting from repeated subcultures, the production of medicines obtained by microbial culture is best based on a system of banked master and working seed lots. From this phage seed lot, a phage API is produced according to a monograph. A Belgian Approved Laboratory performs External Quality Assessments to evaluate the API’s properties and quality. Each batch of these phage APIs will have a batch record, which describes the production process for that batch in detail. Phage APIs can be produced by both private companies and public institutions. The phage API, accompanied by its batch record protocol and the results of the External Quality Assessments, is then transferred to the hospital pharmacy for possible incorporation in magistral formulas. Ideally, active phage APIs are selected against the target bacteria. In comparison to an antibiogram (to test antibiotic sensitivity), as it were, a “phagogram” is performed. Today, no formal guidelines exist with regard to the clinical use (e.g., medical indications, formulations and posology) of magistral phage medicines. However, it is the intention to draft these guidelines as quickly as possible, at the Belgian level and possibly at the European level.

### 3.3. Phage API Monograph

Next, experts of the Queen Astrid military hospital in Brussels, the FAMHP and the Belgian Scientific Institute of Public Health elaborated a pragmatic supplier monograph for phage APIs with a limited use (to hospital pharmacies) status. This document was conceived as a general (applicable to most phages) and evolving document. On 10 January 2018, version 1.0 of the monograph (Supplementary Document 1) received a formal positive advice by the FAMHP.

### 3.4. Pricing and Reimbursement

In terms of pricing, the total cost of a magistral preparation is a reflection of the costs for the products in the preparation, eventually the costs of the prescribed excipients or recipients and an honorarium for the pharmacist for the magistral preparation. Reimbursement of a magistral preparation in Belgium is subject to several criteria: (1) the pharmacist receives a prescription from a physician; (2) this pharmacist makes the magistral preparation and delivers it; (3) products in the magistral preparation are listed on a predefined list of products eligible for reimbursement; and (4) the conditions for reimbursement need to be respected. Bacteriophages are at the moment not listed as products eligible for reimbursement. Therefore, depending on the ultimate price set for a phage magistral preparation, this might (or not) influence the access of phage therapy to patients.

## 4. Conclusions

It seems to be a matter of time before phage therapy regains its status as an established antibacterial tool. However, this will not only depend on the credibility of “phage researchers”, but also on the political context in which they are working. Phage therapy is not sustainable without reimbursement of the researchers providing the therapeutic phages, and so far, phage research is underfunded. Just as drug companies are allowed to profit for some time after developing a drug, there must be some form of compensation for the investigators isolating, characterizing, and optimizing the phages that will be included in future therapeutic phage banks, all before a pharmacist gains access to them for combination. Phage researchers should not be expected to automatically be “altruists”, and compensation must be given for their efforts at developing phage therapy as a medicine.

Believing that Belgium could do some pioneering work in the phage therapy field, the Belgian Minister of Public Health and the FAMHP opened the door to phage medicines that take into account the unique characteristics of phages and the need for personalized “sur-mesure” and sustainable phage therapy approaches [7]. There is every reason to believe that the resulting Belgian “magistral phage medicine” framework will be flexible enough to exploit and further explore the specific nature of phages as co-evolving antibacterials whilst giving precedence to patients’ safety. Importantly, this Belgian solution avoids the application of certain medicinal product requirements that restrain flexible phage therapy approaches, such as compliance to Good Manufacturing Practice (GMP). There are indications that other (EU) countries might also adopt this phage therapy framework in the near future, in anticipation of a European solution. Recently, the biological master file concept was put forward as a European solution to overcome the regulatory challenges of personalized medicines in general and phage medicines more specifically [11].

## Supplementary Materials

The following are available online at http://www.mdpi.com/1999-4915/10/2/64/s1, Document 1. Phage API monograph (version 1.0).
