# Supplementary material for: The Magistral Phage
Source: Viruses. 2018 Feb 6;10(2):64. doi: 10.3390/v10020064 (PMC5850371; doi:10.3390/v10020064)
Supplement: Supplementary file 1 [file viruses-10-00064-s001.pdf]

PHAGE ACTIVE PHARMACEUTICAL INGREDIENTS

DEFINITION

Phage active pharmaceutical ingredients (APIs) are pharmaceutical raw materials containing naturally occurring bacteriophages (phages in short), which are viruses that infect bacteria. Phages are composed of proteins that encapsulate a DNA or RNA genome, and may have relatively simple or elaborate structures. Phages replicate within a bacterium following the injection of their genome into its cytoplasm.

Phage APIs are intended for use as active ingredients of phage magistral preparations for *in vivo* treatment of bacterial infections (phage therapy).

Phage APIs are available as aqueous physiological solutions containing natural lytic phages (e.g., saline or glucose solutions) that may contain a buffer or as dried or freeze-dried powder. As active ingredients of magistral preparations, they are intended to be diluted or reconstituted and/or combined with the necessary excipients, in a hospital pharmacy officina, immediately before use on a named patient basis. Dosage forms may consist of capsules, creams, ointments, liquid preparation for oral use, cutaneous application, inhalation or parenteral administration, etc. The excipients needed to formulate these dosage forms must allow the required phage activity during the intended application period.

Each phage API contains one phage strain and various phage strains APIs may be combined into one magistral preparation to broaden the spectrum of activity of the medicine.

The magistral preparation of phage therapy products is a practical way for medical doctors to personalize antibacterial treatments.

This monograph does not apply to phage derived products such as phage endolysins. It does not necessarily apply to phage products for veterinary use or for decontamination purposes.

In addition to the requirements specified in this general monograph, specific requirements for production, in process testing and release testing might be included in individual monographs.

PRODUCTION

MANUFACTURING PROCESS

Phage APIs are generally obtained by propagation in host bacterial strains and are purified using appropriate methods shown to preserve the biological properties of the phages. Phage APIs are manufactured under conditions designed to minimise microbial contamination and phage degradation.

Purification procedures need to be designed to minimize the content of harmful bacterial or culture medium components (e.g., bacterial endotoxins and animal products).

The manufacturing process must be described in detail (equipment, materials, culture media, additives, culture conditions, purification steps,..) in standard operating procedures (SOPs) and must be validated to confirm that the process can reliably output phage APIs of a determined standard.

The following manufacturing process has shown to be suitable for the small-scale production of qualitatively acceptable and safe phage APIs. It is indicative and based on the state of the art and available knowledge from peer reviewed scientific literature.

The manufacturing process comprises various stages.

De novo **phage isolation.** Natural phages are generally isolated from environmental samples such as sewage and river water or from clinical samples. Usually, the sample, culture medium and phage sensitive host bacteria (typically 10^7-10^8 colony forming units (cfu)) are mixed in a sterile container and incubated under appropriate conditions (typically at 37°C for 1-3 h). If justified, a small volume of chloroform is added and the container is further incubated at 4°C for a short period of time (typically for 1 h). Host bacteria are removed using membrane filtration (0.2-0.5 µm) or by centrifugation. Usually, phages are isolated on bacteriophage sensitive bacteria following the 'double agar overlay method'. Phage lysate is mixed with lukewarm (typically 45°C) culture medium containing 0.5-1% agar and a suspension of bacteriophage sensitive host bacteria (typically 10^7-10^9 cfu/ml) in a sterile container. This mixture is transferred to a sterile cell culture container with culture medium containing 1-3% agar and incubated under appropriate conditions (typically at 37°C for 12-36 h). The resulting plaques ('clear' zones formed in a lawn of bacterial cells due to lysis by phages) with different morphology are transferred to sterile culture media in sterile containers and incubated under appropriate conditions (typically at 37°C for 1-3 h). If justified, a small volume of chloroform is added and the containers are further incubated at 4°C (typically for 1 h). For each container, a dilution series (typically log(0) - log(-8)) is made in sterile containers filled with culture medium. A part from each dilution is mixed with lukewarm (typically 45°C) culture medium containing 0.5-1% agar and a suspension of bacteriophage sensitive host bacteria (typically 10^7-10^9 cfu/ml) in a sterile container. This lysate mixture is transferred to cell culture containers with culture medium containing 1-3% agar and incubated (typically at 37°C for 12-36 h). Plates showing 1-10 plaques are visually analysed. Again, all plaques with different morphology are transferred to sterile culture medium in sterile containers and incubated (typically at 37°C for 1-3 h). This complete cycle is repeated until phage lysates with one plaque morphotype, containing one phage clone, are obtained (homogeneous plaques).

If warranted, phages can be incited to evolve in vitro *to exhibit broader host range or higher lytic activity under physiological conditions (e.g., temperature and pH).*

Phage seed lots. Phage seed lots are usually prepared using a slightly modified double-agar overlay method. If justified, another adequate solidifying agent than agar can be used. Monoclonal phage lysate (typically containing 10^3-10^5 plaque forming units (pfu)) is mixed with lukewarm (typically 45°C) culture medium containing 0.5-1% agar and a suspension of phage sensitive host bacteria (typically 10^7-10^9 cfu/ml) in a sterile container. This mixture is transferred to a sterile cell culture container with culture medium containing 1-3% agar and incubated (typically at 37°C for 12-36 h). If justified, a small volume of chloroform is added and the container is further incubated at 4°C (typically for 1 h). The top agar layer is recuperated and transferred to a sterile container. *Alternatively, buffer solution is added to the top agar layer. The cell culture container is shaken (typically for 1-3 h) and the buffer solution is recuperated.* Bacterial cells and cell debris are removed, usually by centrifugation (e.g., 20 min at 6 000*g*) followed by membrane filtration (0.2-0.5 µm). Phage seed lots can be stored using validated preservation/storage (cooling, cryopreservation, freeze-drying,..) methods.

Phage APIs. Phage APIs are prepared in the same way as phage seed lots, but starting from characterised and quality controlled phage seed lots instead of phage lysates. If justified, other agreed manufacturing methods can be used. In addition, bioburden as well as the levels of impurities, including endotoxins (especially for Gram negative host bacteria) are minimized using appropriate methods (e.g.

dedicated filters, affinity columns, tangential flow filtration, cesium chloride banding).

QUALITY SYSTEM AND PRODUCTION ENVIRONMENT

Phage seed lots and phage APIs should be manufactured under a quality system.

The manufacturing of phage APIs from phage seed lots takes place in an environment with specified air quality and cleanliness to minimize the risk of contamination. The effectiveness of these measures is validated and monitored. Where phage APIs are exposed to the environment during processing, without a subsequent microbial inactivation or removal process, an air quality with particle counts and microbial colony counts equivalent to those of Grade A as defined in the current European Guide to Good Manufacturing Practice (GMP), Annex 1 and Directive 2003/94/EC is required with, if the system is not closed, a background environment at least equivalent to GMP Grade B in terms of particles and microbial counts. The biosafety level (BSL) is determined by the host bacteria used in the production processes (e.g., BSL-2 for *Pseudomonas aeruginosa*).

EQUIPMENT AND MATERIALS

All equipment and material are designed and maintained to suit its intended purpose and must minimize any hazard to recipients and staff. All critical equipment and technical devices are identified and qualified, regularly inspected and preventively maintained in accordance with the manufacturers' instructions. Where equipment or materials affect critical processing or storage parameters (e.g., temperature, pressure, particle counts and microbial contamination levels), they must be identified and subjected to appropriate monitoring, alerts, alarms and corrective action, as required, to detect malfunctions and defects and to ensure that the critical parameters are maintained within acceptable limits at all times. All equipment with a critical measuring function is calibrated against a traceable standard if available. Maintenance, servicing, cleaning, disinfection and sanitation of all critical equipment are performed regularly and recorded accordingly.

SOPs detail the specifications for all critical materials and reagents. In particular, specifications for culture media, additives (e.g., solutions) and packaging materials are defined. Where applicable, reagents and materials meet compendial requirements and/or documented specifications and the requirements of Regulation 2017/745 of the European Parliament and of the Council of 5 April 2017 on medical devices and Regulation 2017/746 of the European Parliament and of the Council of 5 April 2017 on *in vitro* diagnostic medical devices. Animal component free culture media and additives should preferably be used. If materials of human or animal origin are used, measures to control endogenous and adventitious agents including transmissible spongiform encephalopathy (TSE) agents should be implemented. Material from TSE-relevant animal species should be subjected to a risk assessment to demonstrate that TSE risk factors have been considered and that the risk has been minimized by applying the principles described in the general text Ph. Eur. 5.2.8 and the Note for Guidance on Minimizing the Risk of Transmitting Animal Spongiform Encephalopathy Agents via Human and Veterinary Medicinal Products (EMEA/410/01). Compliance to these requirements may be demonstrated on production of a TSE Certificate of suitability granted by the EDQM.

Host bacteria used in the manufacturing process are as safe (or least pathogenic) as possible. Non-lysogenic bacterial strains are used, if possible.

TESTS

Various tests can be applied, but validated tests measuring the identity and quantity of phages, bioburden, bacterial endotoxin levels, pH and, where relevant, water content and residual chloroform of phage APIs, performed by a Belgian Approved Laboratory, are mandatory.

HOST BACTERIA

Identification. State of the art clinical microbiology techniques.

PHAGE SEED LOTS

Phage identification. State of the art DNA or RNA sequencing and genome analysis. When reliable *in silico* morphology prediction is not possible, phage morphology should be determined by electron microscopy.

Phage enumeration. The phage enumeration of the phage seed lot should be determined using an appropriate method (e.g. pfu determination, qPCR).

Phage purity. Absence of adventitious agents (e.g., other phages, bacteria, viruses) should be demonstrated using an appropriate method, unless otherwise justified (e.g. virus testing may be omitted if no or only autoclave-sterilized material of human or animal origin is used).

Detection of genetic determinants conferring toxicity, virulence, lysogeny and antibiotic resistance. State of the art DNA or RNA sequencing and genome analysis.

Raw sequencing data must be provided in a broadly accepted format (e.g. FASTQ) to a Belgian Approved Laboratory for review and approval.

PHAGE APIs

All tests are performed under appropriate quality standards (e.g. ISO17025).

Phage identification. The phage strain of a phage API is determined using a validated or qualified phage identification test (e.g. specific PCR, qPCR..)

Quantitative assessment of phages. The potency of the phage API is determined using a validated or qualified assay (e.g. phage-specific qPCR).

Quantitative bioburden determination (EP 2.6.12). The total aerobic microbial count is determined using the official Ph.Eur. method or, where justified and authorised, using a validated alternative method. Phage APIs are required to contain ≤ 10 cfu/100 ml or g.

Bacterial endotoxins (EP 2.6.14). The test for bacterial endotoxins is used to detect and quantify endotoxins of gram-negative bacterial origin using amoebocyte lysate from horseshoe crab (*Limulus polyphemus* or *Tachypleus tridentatus*). The endotoxin concentration in the phage API should remain below the endotoxin threshold specified in the individual monograph. The endotoxin limit depends on the final therapeutic product (magistral preparation) and its route of administration and is stated in the individual monograph according to compendial requirements. *The maximal dose administered by the intended route per hour should not contain sufficient endotoxin to cause a toxic reaction. For instance, as stated in EP 5.1.10, the maximum dose for intravenous injection is 5 Endotoxin Units (EU)/kg/h.*

Potentiometric determination of pH (EP 2.2.3). The pH should conform to the pH specifications set forth in the individual monograph, usually 6.0-8.0 pH.

Water content (EP 2.5.12 or 2.5.32). Dried phage APIs are tested for water content. The maximum water content is 3.0 per cent m/m, unless otherwise stated in specific monograph (e.g. APIs intended for oral lyophilisates).

Impurities. Process-related impurities should be quantified

and qualified. In particular, when chloroform or any other particular reagent is used in the manufacture of the phage API, an appropriate validated procedure is to be employed for the quantification of the residual chloroform/reagent. Appropriate acceptance criteria should be set up such that the amounts of chloroform/reagent intake are consistently below levels that are demonstrated to be safe.

STORAGE

Phage APIs should be stored under the conditions specified in the individual monograph.

SHELF LIFE

Phage quantity using a stability indicative method, bioburden, pH and, where relevant, water content are periodically determined. The shelf life is the time period during which phage quantity, bioburden, pH and, where relevant, water content of the API remain within the limit thresholds specified in the individual monograph.

LABELLING

The label states:

- the identity and quantity of the phages within the API;
- the type of species and strain of host bacteria used as a substrate for the production of the phage API;
- the type of species and strains of bacteria that the phages are able to lyse;
- the storage conditions;
- the production date;
- the expiration date;
- for dried preparations:
  - the name, composition and volume of the reconstituting liquid to be added;
  - the period of time within which the preparation is to be used after reconstitution;
- instructions for reporting serious adverse reactions and/or events;
- instructions how to dispose of unused (expired) bacteriophage products.

SURVEILLANCE

The clinical use of phage API based magistral preparations must be surveyed and reported, including possible adverse events and reactions associated with their use. A centralized reporting system and a register for therapeutic phage applications are warranted.
